# Trunk Alignment in Physically Active Young Males with Low Back Pain

**DOI:** 10.3390/jcm11144206

**Published:** 2022-07-20

**Authors:** Magdalena Plandowska, Agnieszka Kędra, Przemysław Kędra, Dariusz Czaprowski

**Affiliations:** 1 Faculty of Physical Education and Health, Jozef Pilsudski University of Physical Education in Warsaw, 21-500 Biala Podlaska, Poland; agnieszka.kedra@awf.edu.pl (A.K.); przemyslaw.kedra@awf.edu.pl (P.K.); 2 Department of Health Sciences, University College in Olsztyn, 10-283 Olsztyn, Poland; dariusz.czaprowski@interia.pl; 3 Center of Body Posture, Bydgoska 33, 10-243 Olsztyn, Poland

**Keywords:** low back pain, physical activity, trunk alignment, rasterstereographic system, young males

## Abstract

Background: Systematic physical activity has become an essential part of the guidelines for the prevention and treatment of low back pain (LBP). The aim of this study was to assess differences in trunk alignment parameters with regard to the level of physical activity in groups of individuals with and without LBP. Methods: 43 participants with LBP and 37 healthy persons were recruited. Participants were divided into two subgroups: (1) students with a moderate level of physical activity (MPA); (2) students with a high level of physical activity (HPA). An original questionnaire was used to assess the prevalence of LBP. The spinal posture was measured using the Formetric 4D rasterstereographic system. Results: There were no significant differences between groups for any of the parameters assessed: trunk imbalance, trunk inclination, trunk torsion, pelvic tilt, pelvic inclination, pelvic torsion, kyphotic angle and lordotic angle. Conclusions: There are no differences in trunk alignment parameters in the sagittal, frontal and transversal planes between physically active males with and without LBP. Therefore, it can be assumed that physical activity may reduce the risk of the deterioration of trunk alignment in males with LBP younger than 25 years.

## 1. Introduction

The ability to maintain an upright posture is fundamental to normal activities of daily living. The normal curvatures of the spine in the sagittal plane (cervical lordosis, thoracic kyphosis and lumbar lordosis) are balanced with each other in normal upright posture [1], while abnormal curvatures of the spine (sagittal imbalance) cause increased muscular effort and energy expenditure, causing pain, fatigue, and disability [2]. It has been reported in many disorders, especially low back pain (LBP). The studies suggest that people with LBP often have disturbances in the motor control of deep trunk muscles [3,4,5,6,7].

There is a lack of literature that analyses and compares the difference in spinal posture in the LBP population with healthy counterparts considering the level of physical activity. The recent Lancet low back pain series recommended exercises [8]. Physical activity is effective in the prevention and treatment of LBP [9]. Physical activity is significant to improving functional activity, influences bone modeling, helps to strengthen the muscles and prevents a reduction in postural stability [10,11]. This is very important for all people, especially individuals with LBP. On the other hand, a high level of physical activity might increase the risk of LBP prevalence. Heneweer et al. [12] described the association between physical activity and LBP as a U-shaped curve—inactivity and over-activity are harmful to the health of the spine.

The influence of a low level of physical activity on body posture is known, while the potential impact of a high level of physical activity in people with LBP has received less attention to date. Therefore, the aim of this study was to assess differences in trunk alignment parameters with regard to the level of physical activity in groups of individuals with and without LBP.

## 2. Materials and Methods

### 2.1. Participants

Eighty university students of a Bachelor course in Physical Education participated in this cross-sectional study, including 43 individuals with a history of LBP and 37 healthy persons without LBP. Based on the sample size (*n* = 80), a power analysis was set at 0.75. The inclusion criteria for the LBP group were as follows: (a) male; (b) age between 20 and 23 years; (c) experiencing LBP for the last year; (d) reporting an average low back pain intensity of 4 or greater as measured by the Visual Analogue Scale (VAS; 4–10). Participants were excluded from the study if they: (a) were experiencing very rare pain; (b) had any neurological, cardiovascular, rheumatic or vestibular disorders; (c) had conditions that could interfere with the measure of the Formetric Diers such as back tattoos or prostheses; (d) were experiencing low back pain at the time of the examination. Healthy individuals had no history of LBP within the last year. The demographic characteristics of the participants are presented in Table 1.

Participants were divided into two subgroups, i.e., (1) students with a moderate level of physical activity (MPA) and (2) students with a high level of physical activity (HPA). The inclusion criteria in the MPA group were as follows: (a) attending physical education classes included in the curriculum; (b) taking up leisure-time physical activity no more than once per week and no longer than 60 min. The inclusion criteria in the HPA group were as follows: (a) attending physical education classes included in the curriculum; (b) training a minimum of 90 min per day—5 times per week; (c) training experience—a minimum of 3 years. The HPA group included students who trained handball or volleyball.

All the participants gave their written informed consent. The study was conducted in accordance with the Declaration of Helsinki, and the research was accepted by the Senate Scientific Research Ethics Commission.

### 2.2. Questionnaire

A questionnaire was used to assess the prevalence of LBP [13]. The first page of the questionnaire included an explanation of the study purpose, instructions and questions on age, body mass and body height.

The next page of the questionnaire included questions related to:

(a) training (sport)—sports discipline, number of training days per week, number of training hours per day;

(b) experiencing LBP within the last year (12 months). Individuals who responded positively (“yes”) to question “Have you experienced low back pain for the last year (12 months)?” answered the question in the second part. Individuals who responded negatively (“no”) to this question were asked not to answer the remaining questions.

The second part of the questionnaire included questions on the frequency and intensity of LBP, the types of situation in which LBP occurred or increased.

The Visual Analogue Scale (VAS) was used to assess average pain intensity. Participants were asked to rate their maximal pain intensity from the last year on a 10 cm line. The centimetres marked by the participants were measured and classified according to the following key: 0—no pain, 1–3—mild pain, 4–6—moderate pain and 7–10—severe pain [14].

In this study, we defined LBP as the pain localized below the costal margin and the inferior gluteal folds without sciatica, with an average pain intensity of 4 or greater (moderate or severe pain), as measured by the Visual Analogue Scale (VAS).

Information about musculoskeletal disorders was also obtained from systematic medical examinations conducted by a sports doctor.

### 2.3. Spine Shape Evaluation

The spinal posture was measured using the Formetric 4D rasterstereographic system (DIERS, International GmbH, Schlangenbad, Germany). It is a valid and reliable method used for three-dimensional analysis of the spine [15,16].

Prior to the study, each participant was given detailed information on the testing procedures and research methodology.

It was assumed that the participants experiencing low back pain at the time of the examination would adopt a non-habitual body posture which may be accompanied by an unnatural positioning of body parts. Therefore, the participants were asked whether they felt pain at the time of taking the measurement. None of the participants reported pain when they were tested.

Participants removed all clothing except for a pair of shorts. The subject’s back was exposed from the beginning of the intergluteal fissure to the occiput. Subjects were placed barefoot in a comfortable standing position, with their knees extended and their arms resting naturally alongside their hips. To standardize the subjects’ positioning, a horizontal line was drawn on the floor to provide a reference for their heels. Participants were positioned 2 m from the Formetric 4D projection and camera unit. The unit projected stripes of light on the surface of the participant’s back. In accordance with the recommendations of Guidetti et al. [17], no reflective markers were positioned on participants.

Each scan was completed in the DIERS data collection and processing software. During each scan, 12 images were recorded over the 6 s (2 Hz). Each scan was processed as per the manufacturer’s instructions. On each of the collected images, the software automatically indicated the location of the left (DL) and right (DR) sacral dimples associated with the posterior superior iliac spine and the location of the vertebral prominens (VP). The middle point between the dimples (DM) was determined from the location of DL and DR.

The test was performed by the same examiner. Participants were examined individually. They were supplied with the same instructions before the test.

**Figure 1:**
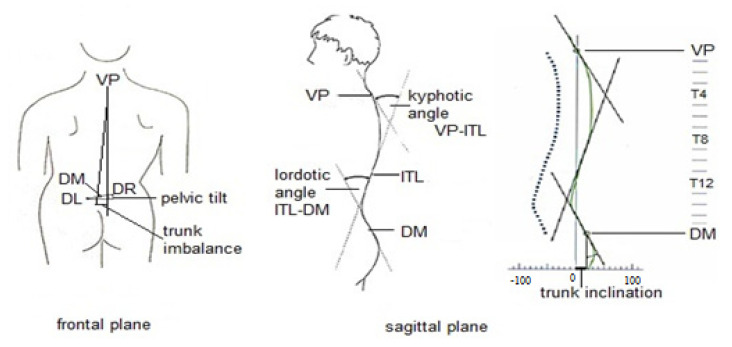
Trunk alignment parameters, measured with Diers Formetric. Abbreviations: VP—vertebra prominens; DM—midpoint between the left and right sacral dimples; DL—left sacral dimple; DR—right sacral dimple; ITL—thoracic-lumbar inflection point.

The following trunk alignment parameters were analysed:

Trunk and pelvic parameters: trunk imbalance VP-DM [mm], trunk inclination VP-DM [mm], trunk torsion [°], pelvic tilt DL-DR [mm], pelvic inclination DL-DR [°] (dimples), pelvic torsion DL-DR [°];

Spinal curve angles: kyphotic angle VP-ITL [°], lordotic angle ITL-DM [°] (Table 2, Figure 1).

All the tests were performed during morning hours in the laboratory room at the Regional Centre for Research and Development.

### 2.4. Statistical Analysis

The parameters were described using basic descriptive statistics measurements, i.e., percentage for qualitative variables, mean and standard deviation for quantitative variables. Descriptive statistics were calculated separately for both LBP and healthy groups. The chi-square test was used for categorical variables (the prevalence and frequency of LBP with regard to the level of physical activity). The Shapiro–Wilk test was used to check the compliance of the results with normal distribution. The data were analysed using a two-factor ANOVA. The between-subject factors were group (LBP and healthy) and the level of physical activity (MPA and HPA). Statistical significance was set at *p* < 0.05. The collected material was organised and analysed using Statistica 13.3 calculation software.

## 3. Results

LBP was common among participants with a HPA than among their peers with a MPA (59.1% vs. 47.2%, respectively). The difference was not significant (*p* = 0.29) (Table 3).

The largest group was those who experienced LBP a few times a year (3–6/year) (60.5%). Participants with HPA declared frequent or constant pain more often than their peers with MPA (42.3% vs. 35.3%, respectively). The difference was not statistically significant (*p* = 0.64). The analysis of LBP intensity showed that 62.8% of the participants declared moderate pain. Nearly 40% of the participants limited their physical activity when the pain was very intensive. In 46.5% of participants, LBP intensified during physical activity, more often among the participants with HPA than among students with MPA (57.7% vs. 29.4%, respectively) (Table 4).

The analysis of trunk alignment revealed no differences in trunk alignment parameters in the sagittal, frontal and transversal planes between active participants with and without LBP regardless of the level of physical activity (Table 5 and Table 6).

## 4. Discussion

The aim of this study was to assess differences in trunk alignment with regard to the level of physical activity in groups of individuals with and without LBP. Our results revealed that no differences were found in trunk alignment parameters in active males with LBP compared with healthy individuals. Similar findings were reported in other studies [18,19]. A systematic review by Laird et al. [18] showed that people with LBP display no difference in their lordosis angle and pelvic tilt angle in standing. A study conducted by Tatsumi et al. [20] indicates that participants with LBP have a large anteversion of the pelvic angle. Authors suggested that large lumbar lordosis is not associated with LBP. Other studies showed that participants with LBP are characterized by a loss of lumbar lordosis and an increase of pelvic tilt [21,22,23,24]. A systematic review and meta-analysis demonstrate a strong relationship between LBP and decreased lumbar lordotic curvature [24]. Moreover, Barrey et al. [22,23] showed that participants with chronic LBP and lumbar degenerative disease are characterized by sagittal imbalance. Our study did not observe a significant difference in pelvic asymmetry parameters between participants with and without LBP. Other studies showed that pelvic asymmetry is unlikely to be associated with the prevalence of LBP [25,26].

Studies based on the analysis of the relation between physical activity and LBP reported that a moderate level of physical activity is associated with lower prevalence of LBP [9,27]. Another study found that there is strong evidence that a heavy physical workload is a risk factor for back pain [28]. LBP is common in the athletic population [29]. Although it is well known that physical activity can reduce risk for musculoskeletal diseases by improving muscle strength, bone metabolism and functional health [10,11], there is a lack of literature that analyses and compares the difference in spinal posture in the LBP population considering the level of physical activity. Therefore, the aim of this study was to assess differences in trunk alignment parameters with regard to the level of physical activity in groups of individuals with and without LBP. The results of our study showed that there are no significant differences in trunk alignment parameters in the sagittal, frontal and transversal planes between active males with and without LBP, regardless of the level of physical activity. Despite the fact that a high level of physical activity might increase the risk of LBP prevalence, this study showed that physical activity may reduce the risk of the deterioration of trunk alignment in males with LBP younger than 25 years. Our findings are consistent with recommendations from LBP management. Prevention and treatment guidelines recommend staying active and continuing with normal activities. Exercise can be utilised as a central component of treatment. Recommended physical treatments include a graduated activity or exercise programme that targets the improvement of function and prevention of worsening disability [8]. Exercise is of great importance to improve functional ability and health-related quality of life in patients with LBP. Being active is associated with less pain, including LBP, and injury, as long as the rate of increase in activity is managed appropriately and other important factors are also taken into account (e.g., sleep, mood, relationships) [30].

### 4.1. Limitations

The present study is limited by its cross-sectional design; therefore, causality cannot be inferred. The characterization of pain was not our aim; therefore, the detailed characterization of pain has been omitted. Another limitation was a small number of study participants. The research was planned with all students in the 1st, 2nd and 3rd year of PE on a day of testing. The target group was smaller due to the fact that this was during the COVID-19 pandemic. Moreover, different sports were considered together. It is worth continuing the observation with a larger athlete sample size to verify the observed results, with a view to identifying differences between sports.

### 4.2. Study Strenghts

According to the authors’ knowledge, it is the first study to compare differences in trunk alignment parameters taking into account different levels of physical activity (MPA and HPA). The spinal posture was measured using a valid and reliable method used for three-dimensional analysis of the spine (the Formetric 4D rasterstereographic system, DIERS) [15,16]. However, the study showed that reliability of trunk imbalance and pelvic torsion is lower than the overall excellent reliability of sagittal plane parameters [16]. Prevalence of LBP was measured using a reliable questionnaire, and the Kappa coefficient value for all the analysed variables was equal to or higher than 0.93 [13].

## 5. Conclusions

There are no differences in trunk alignment in the sagittal, frontal and transversal planes between physically active males with and without LBP, regardless of the level of physical activity. Therefore, it can be assumed that physical activity may reduce the risk of the deterioration of trunk alignment in males with LBP younger than 25 years.

## Figures and Tables

**Table 1:** Characteristics of study population (*n* = 80).

Characteristics	LBP *n* = 43 Mean (SD)	Healthy *n* = 37 Mean (SD)
Age (years)	21.2 (0.8)	21.4 (0.9)
Weight (kg)	81.2 (9.1)	79.8 (9.6)
Height (cm)	183.2 (7.7)	181.1 (5.4)
BMI (kg/m^2^)	24.6 (3.5)	24.0 (2.6)

**Table 2:** Trunk alignment parameters and their description.

Trunk Alignment Parameters	Description
Trunk imbalance VP-DM [mm]	The lateral deviation of VP from DM
Trunk inclination VP-DM [mm]	A difference in height between VP and DM, based on a vertical plane
Trunk torsion [°]	The torsion of the surface normals of DM and VP
Pelvic tilt DL-DR [mm]	The difference in height of the DL and DR
Pelvic inclination DL-DR [°] (dimples)	The mean torsion of the DL and DR surface normals
Pelvic torsion DL-DR [°]	The torsion of the surface normals on DL and DR
Kyphotic angle VP-ITL [°]	The angle between VP and the thoracic-lumbar inflection point ITL
Lordotic angle ITL-DM [°]	The angle between the surface tangents of the thoracic-lumbar inflection point ITL and the lower lumbar-sacral inflection point ILS

VP—vertebra prominens; DM—midpoint between the left and right sacral dimples; DL—left sacral dimple; DR—right sacral dimple; ITL—thoracic-lumbar inflection point.

**Table 3:** The prevalence of LBP.

			MPA *n* = 36	HPA *n* = 44	*p* Value
**LBP**	*n* = 43	n (%)	17 (47.2)	26 (59.1)	0.29
**Healthy**	*n* = 37	n (%)	19 (52.8)	18 (40.9)	

LBP—low back pain; MPA—moderate level of physical activity; HPA—high level of physical activity. Statistical significance was set at *p* < 0.05.

**Table 4:** The frequency and intensity of LBP, the influence of LBP on the undertaken PA.

		All *n* = 43	MPA *n* = 17	HPA *n* = 26	*p* Value
**LBP frequency**					
LBP a few times a year (3–6/year)	*n* (%)	26 (60.5)	11 (64.7)	15 (57.7)	0.64
Frequent or constant LBP (more than 1–2 months)	*n* (%)	17 (39.5)	6 (35.3)	11 (42.3)	
**LBP intensity**					
Moderate	*n* (%)	27 (62.8)	10 (58.8)	17 (65.4)	0.66
Severe	*n* (%)	16 (37.2)	7 (41.2)	9 (34.6)	
**The influence of LBP on the undertaken PA**					
No influence	*n* (%)	25 (58.1)	10 (58.8)	15 (57.7)	0.94
I limit the amount of PA when the pain is very intensive.	*n* (%)	16 (37.2)	7 (41.2)	11 (42.3)	
**A higher intensity of LBP during physical exercises**					
No	*n* (%)	23 (53.5)	12 (70.6)	11 (42.3)	0.06
Yes	*n* (%)	20 (46.5)	5 (29.4)	15 (57.7)	

LBP—low back pain; PA—physical activity; MPA—moderate level of physical activity; HPA—high level of physical activity. Statistical significance was set at *p* < 0.05.

**Table 5:** Means and standard deviations for trunk alignment parameters.

		MPA	HPA	All
		M (SD)	M (SD)	M (SD)
**Trunk and pelvic parameters**				
Trunk inclination VP-DM [mm]	LBP	18.4 (15.4)	24.0 (13.8)	21.8 (14.5)
	Healthy	12.9 (10.2)	18.9 (16.7)	15.8 (13.9)
Trunk imbalance VP-DM [mm]	LBP	10.1 (5.8)	9.4 (8.1)	9.7 (7.2)
	Healthy	8.3 (7.1)	7.1 (5.0)	7.7 (6.1)
Trunk torsion [°]	LBP	3.4 (2.6)	4.2 (3.0)	3.9 (2.9)
	Healthy	3.5 (2.2)	3.5 (2.9)	3.5 (2.5)
Pelvic tilt DL-DR [mm]	LBP	4.4 (4.1)	4.7 (3.9)	4.6 (3.9)
	Healthy	4.3 (3.0)	4.0 (1.8)	4.1 (2.4)
Pelvic inclination (dimples) [°]	LBP	16.7 (5.1)	18.4 (4.1)	17.8 (4.6)
	Healthy	17.2 (4.9)	18.4 (4.0)	17.8 (4.4)
Pelvic torsion DL-DR [°]	LBP	2.9 (2.0)	2.3 (1.7)	2.6 (1.8)
	Healthy	2.6 (1.6)	2.5 (1.4)	2.5 (1.5)
**Spinal curve angles**				
Kyphotic angle VP-ITL [°]	LBP	43.5 (8.7)	45.3 (7.2)	44.6 (7.8)
	Healthy	44.4 (6.8)	45.2 (6.5)	44.8 (6.6)
Lordotic angle ITL-DM [°]	LBP	31.3 (6.7)	30.6 (6.2)	30.9 (6.3)
	Healthy	31.3 (7.1)	31.0 (6.9)	31.1 (6.9)

LBP—low back pain; M—mean; SD—standard deviation; PA—physical activity; MPA—moderate level of physical activity; HPA—high level of physical activity; VP—vertebra prominens; DM—midpoint between the left and right sacral dimples; DL—left sacral dimple; DR—right sacral dimple; ITL—thoracic-lumbar inflection point.

**Table 6:** Summary of analysis of variance for trunk alignment parameters.

	Group	Level of PA	Group × Level of PA
**Trunk and pelvic parameters**			
Trunk inclination VP-DM [mm]	F = 2.769 *p* = 0.100	F = 3.275 *p* = 0.074	F = 0.006 *p* = 0.939
Trunk imbalance VP-DM [mm]	F = 1.897 *p* = 0.172	F = 0.351 *p* = 0.555	F = 0.032 *p* = 0.857
Trunk torsion [°]	F = 0.270 *p* = 0.604	F = 0.481 *p* = 0.489	F = 0.518 *p* = 0.473
Pelvic tilt DL-DR [mm]	F = 0.310 *p* = 0.579	F = 0.000 *p* = 0.994	F = 0.122 *p* = 0.727
Pelvic inclination (dimples) [°]	F = 0.052 *p* = 0.819	F = 1.936 *p* = 0.168	F = 0.073 *p* = 0.787
Pelvic torsion DL-DR [°]	F = 0.071 *p* = 0.789	F = 0.745 *p* = 0.390	F = 0.438 *p* = 0.509
**Spinal curve angles**			
Kyphotic angle VP-ITL [°]	F = 0.057 *p* = 0.811	F = 0.638 *p* = 0.427	F = 0.073 *p* = 0.787
Lordotic angle ITL-DM [°]	F = 0.010 *p* = 0.921	F = 0.111 *p* = 0.739	F = 0.020 *p* = 0.888

PA—physical activity; VP—vertebra prominens; DM—midpoint between the left and right sacral dimples; DL—left sacral dimple; DR—right sacral dimple; ITL—thoracic-lumbar inflection point. Group: LBP vs. Healthy; Level of PA: MPA vs. HPA. Statistical significance was set at *p* < 0.05.

## Data Availability

Data are contained within the article or Supplementary Materials.

## Supplementary Materials

The following supporting information can be downloaded at: https://www.mdpi.com/article/10.3390/jcm11144206/s1, supplementary file: Dataset.
